# Measuring Protein–Ligand Binding by Hyperpolarized Ultrafast NMR

**DOI:** 10.1021/jacs.3c14359

**Published:** 2024-02-19

**Authors:** Chang Qi, Otto Mankinen, Ville-Veikko Telkki, Christian Hilty

**Affiliations:** † Chemistry Department, Texas A&M University, College Station, Texas 77843-3255, United States; ‡ NMR Research Unit, Faculty of Science, University of Oulu, 90014 Oulu, Finland

## Abstract

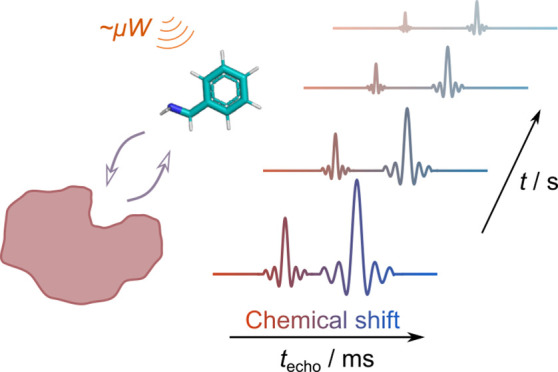

Protein–ligand interactions can be detected by observing changes in the transverse relaxation rates of the ligand upon binding. The ultrafast NMR technique, which correlates the chemical shift with the transverse relaxation rate, allows for the simultaneous acquisition of *R*_2_ for carbon spins at different positions. In combination with dissolution dynamic nuclear polarization (D-DNP), where the signal intensity is enhanced by thousands of times, the *R*_2_ values of several carbon signals from unlabeled benzylamine are observable within a single scan. The hyperpolarized ultrafast chemical shift-*R*_2_ correlated experiment separates chemical shift encoding from the readout phase in the NMR pulse sequence, which allows it to beat the fundamental limit on the spectral resolution otherwise imposed by the sampling theorem. Applications enabled by the ability to measure multiple relaxation rates in a single scan include the study of structural properties of protein–ligand interactions.

Molecular dynamics and interactions are reflected in nuclear spin relaxation parameters, leading to numerous applications of NMR relaxometry in catalysis, protein function and folding, ligand binding and drug discovery, and others.^1,2^ Relaxation rates depend sensitively on molecular correlation times, order parameters, and other model-based parameters, providing a window into motions on the picosecond to nanosecond time scale. Relaxation dispersion, i.e. the measurement of relaxation at different magnetic field strengths or with different spin–echo refocusing rates, further reveals kinetic and chemical exchange processes, such as the binding and unbinding of ligands, on the millisecond time scale.^3^

Nuclear spin hyperpolarization enables the application of NMR in new contexts. Hyperpolarization can provide signal enhancements of thousands of fold or more. NMR spectra can be measured at low concentration and with high time resolution in single scans. Thus, in-operando NMR or the study of biomolecular processes under physiological conditions becomes possible.^4−7^

Typical NMR spectra include chemical shift information in one or more dimensions resolving the individual positions in a molecule. Thus, it is possible to identify flexible or disordered regions in a protein, find binding interfaces, and determine the dynamics of active sites. The capability for real-time acquisition of NMR spectra, frequently the goal of hyperpolarized NMR spectroscopy, unfortunately is inherently limited by the inverse relationship between spectral resolution in indirect dimensions and measurement time. The ability to distinguish molecular sites degrades, in some instances severely, with the requirement for an increased time resolution.

Here, we overcome this limitation for the measurement of spin–spin (*R*_2_) relaxation using single-shot hyperpolarization such as dissolution dynamic nuclear polarization (D-DNP).^8^ The method is based on the principle of two-dimensional ultrafast NMR spectroscopy.^9−11^ An ultrafast NMR pulse sequence (Figure 1) encodes the chemical shift into a spatial dimension of the NMR sample using a combination of pulsed field gradients and adiabatic frequency-swept pulses. The encoded signals are read out in a spin–echo pulse train, also under the application of pulsed field gradients. A similar encoding and detection scheme has been applied to the measurement of *J*-coupling constants^12^ and to enhance sensitivity of indirectly observed heteronuclear spectra by summing together multiple echoes.^13^ Instead of a Fourier dimension, signals with different chemical shifts appear as echoes at different times, whereby the spectrum is directly represented in the acquired time-domain data.^14^ Importantly, the gradient encoded chemical shift dimension fully separates the spectral parameters from the real time of signal acquisition. By selection of the parameters of the encoding gradients in combination with the chirp encoding pulses, the resolution and spectral width are defined independently of the signal acquisition period.

**Figure 1:**
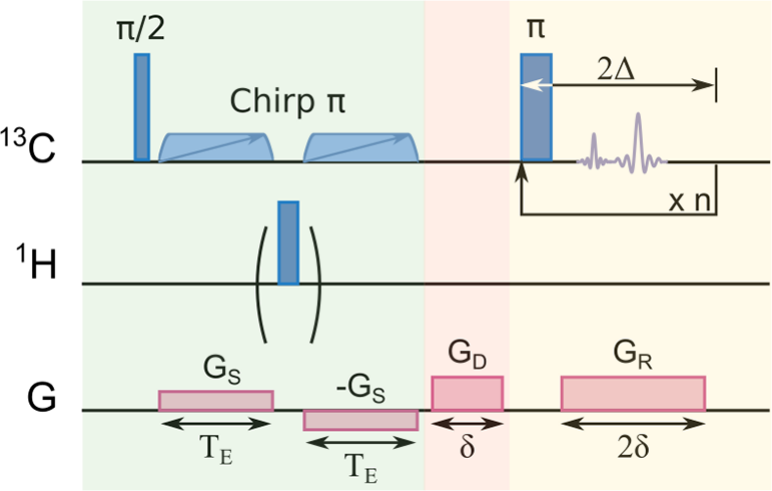
Ultrafast NMR pulse sequence for correlating spatially encoded chemical shift with *R*_2_ relaxation detected in the real-time readout period.

Benzylamine is a ligand for the trypsin protein. D-DNP provided a signal enhancement of 4500–5800 for the aromatic carbon atoms in this molecule (Figures 2a and S1–S2). In general, D-DNP allows for the production of nuclear spin polarization on NMR active nuclei in most small molecules. It facilitates spectroscopy of low-γ nuclei at a much lower concentration than otherwise possible. The direct detection of ^13^C becomes possible, benefiting from the large chemical shift dispersion of this nucleus. We have previously demonstrated that biomolecular interactions such as protein–ligand binding can be characterized by hyperpolarized ^13^C relaxometry,^15^ and show here the substantial advantages offered by the enhanced spectral resolution through ultrafast encoding.

**Figure 2:**
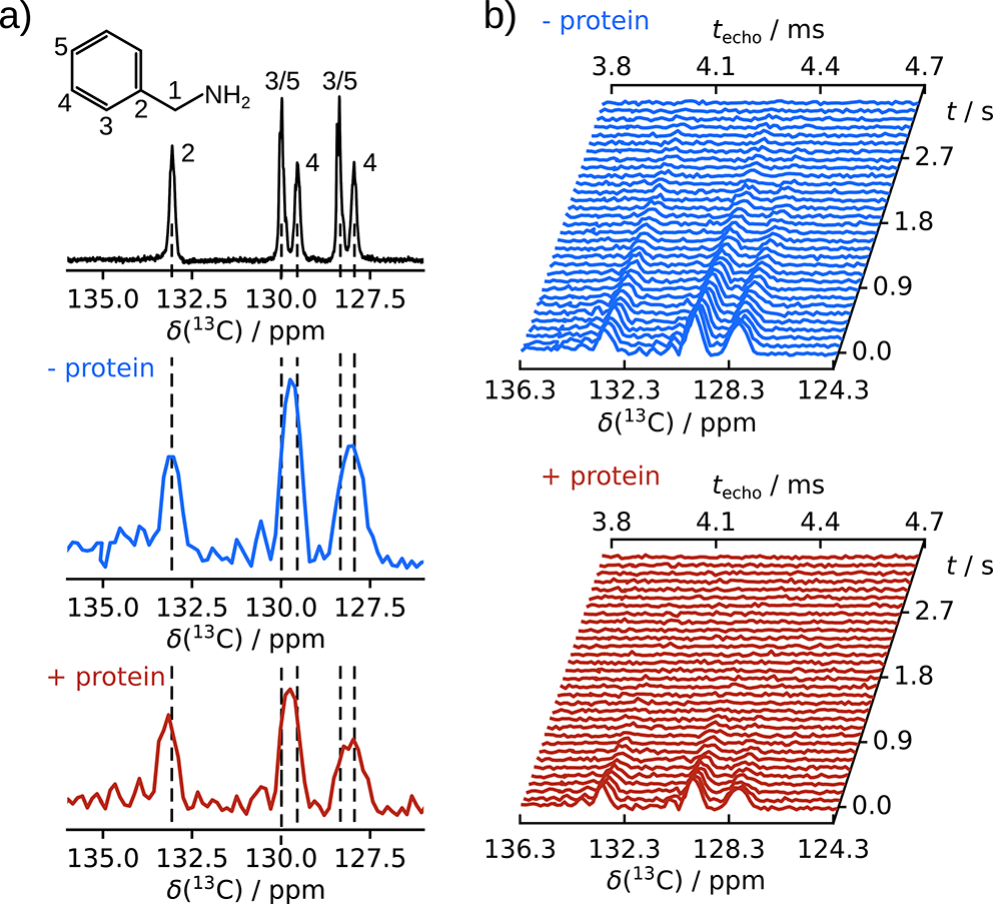
(a) Hyperpolarized ^13^C NMR spectrum of benzylamine without ^1^H decoupling (top). The sum of the magnitude of the first 16 echoes from the ultrafast chemical shift - *R*_2_ correlation experiments of benzylamine in the absence (middle) and presence (bottom) of protein. The *T*_E_ is 5 ms, and the echo time 2Δ is 7 ms. Dashed lines indicate the peak alignment. (b) Ultrafast *R*_2_ measurements of benzylamine in the absence (top) and presence (bottom) of trypsin protein corresponding to the middle and bottom spectra in (a). The final concentration of benzylamine is 6.54 mM in the top ultrafast spectrum and that of benzylamine and trypsin in the bottom spectrum are 6.69 mM and 32.3 μM, all at natural isotope abundance. The spectra are plotted as the sum of the magnitude of 16 successive spin echoes, and the chemical shift axis is calibrated (eq S1 and Figure S3).

In benzylamine, the aromatic carbon signals are closely spaced between 127 and 133 ppm. The assignments are indicated with numbers in Figure 2a. The small chemical shift spacing, despite the use of ^13^C, illustrates the necessity for high resolution in the measurement of *R*_2_ relaxation of this and other biological molecules.

The ultrafast spectra in Figure 2a were measured after injection of the hyperpolarized sample into a flow cell in the NMR spectrometer (Figure S1), with or without admixing of the protein.^16^ Convective motions persisting after injection of samples into conventional NMR tubes can have significant spectroscopic effects.^17^ The motions due to laminar flow in the flow cell exhibit a sufficiently rapid decay that enables the use of pulsed field gradients for encoding.^18^ Here, the experiment was started after a 1.5 s stabilization delay. In the ultrafast experiment, the line width and thus the resolution depend primarily on the duration of the encoding gradient *T*_E_, and notably not on the echo duration. With the experimental parameters used, the theoretical width at half height is calculated to be 60 Hz, corresponding to about 0.6 ppm in the ^13^C spectra (eq S2),^14^ which is in a good agreement with the 0.63 ppm width at half height measured from the C2 peaks (Figure 2a middle and bottom). In contrast, a conventional Carr–Purcell–Meiboom–Gill (CPMG) experiment with the same spin echo time of 7 ms would only achieve a point-to-point spectral resolution of 143 Hz and a width of 172 Hz for the resulting sinc shaped peak (Supporting Information). The peak for C2 is separated from the nearest signal by 5 times the line width, and the doublets of peaks 3/5 and 4 with a separation of 2 ppm are readily resolved. In these spectra, the encoded chemical shift range was selected to include the aromatic carbons.

The *R*_2_ relaxation rates for each observed signal were determined by fitting a single exponential curve to the signal integrals from each echo (Figures 3 and S4). The relaxation rates for the free ligand are 1.01 ± 0.20 s^–1^, 0.84 ± 0.02 s^–1^, and 0.98 ± 0.04 s^–1^ for the signals at 133.1, 129.7, and 127.9 ppm. The same experiments performed with admixing of ∼30 μM trypsin protein to the hyperpolarized sample resulted in significantly larger *R*_2_ relaxation rates of 2.39 ± 0.42 s^–1^, 1.97 ± 0.08 s^–1^, and 2.67 ± 0.06 s^–1^. This difference demonstrates that the ligand is binding to the protein. *R*_2_ values measured in the absence or presence of protein are averaged from three repetitions (Table S1).

**Figure 3:**
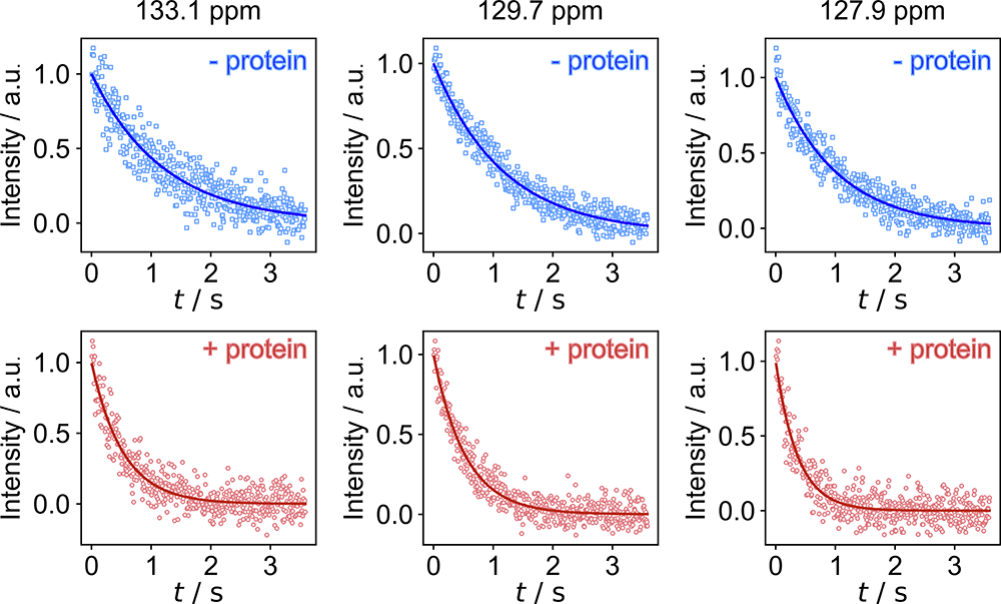
Signal integrals with exponential fit for determining *R*_2_ relaxation (normalized). Relaxation rates in the free ligand experiment (top) are 0.83 ± 0.05 s^–1^, 0.86 ± 0.03 s^–1^, 0.98 ± 0.03 s^–1^ and in the ligand with protein experiment (bottom) are 1.90 ± 0.07 s^–1^, 1.88 ± 0.05 s^–1^, 2.73 ± 0.11 s^–1^ for the signals at 133.1, 129.7, 127.9 ppm, respectively.

The relaxation rates measured with the ultrafast experiment are larger than those in conventional CPMG because of the pulsed field gradients during the signal acquisition.^19^ The observed relaxation rate is the sum of the intrinsic relaxation rate and a diffusion contribution, *R*_2,obs_ = *R*_2,intr_ + *R*_diff_ with *R*_diff_ = *b*_uf_*D*/(2Δ).^20,21^ Here, *b*_uf_ is a function of the gyromagnetic ratio, readout gradient, and echo timing, and Δ is half of the echo time (eqs S6 and S7). For the experiment in Figure 2, *R*_diff_ = 2.2 × 10^8^ m^–2^ · *D*. The diffusion coefficient *D* measured for benzylamine is 0.82 × 10^–9^ m^2^ s^–1^, which results in a diffusion contribution to the observed *R*_2_ on the order of 0.18 s^–1^ for the free ligand. Furthermore, the diffusion effect is not equivalent for spins that refocus at different times.^22^ Therefore, the doublets of peaks 3/5 and 4 in Figure 2a (middle and bottom) are different in shape, and the rightmost peak relaxes faster than others. In general terms, it is suggested to select weak gradients, where the diffusion contribution is smaller than the intrinsic relaxation, although it is also possible to subtract the diffusion contribution based on the estimations described above.^23−25^ It is further noted that residual convective motion causes an increased apparent diffusion coefficient and thus an increased relaxation contribution in this experiment. The use of the flow cell for injecting the hyperpolarized sample alleviates this effect.^16^

The diffusion contribution is smaller for the protein-bound than for the free ligand, which will reduce the maximum observable contrast in *R*_2_ rates.^26^ However, since the ligand is in fast exchange between bound and free forms, with the bound form populated only on the percent level, this effect is minimal.

The spectra and relaxation data in Figures 2 and 3 were measured with 6.54 mM ligand or 6.69 mM ligand and 32.3 μM protein, without any isotope labeling. The signal-to-noise ratio (SNR) of the tallest peak in the ultrafast data set of the ligand (Figure 2a) is 7.45 without protein and 6.34 with protein. The spectral resolution can be improved by simply extending the duration *T*_E_ of the encoding gradient.^14^ Increasing *T*_E_ from 5 to 20 ms allowed the separation of C3/5 and C4 peaks (Figure 4a). The width of the C2 signal became 25 Hz, at the cost of reducing signal sensitivity and with this molecule reaching experimental limits, as described in Supporting Information. Relaxation rates were therefore not further analyzed. The binding of a fast-exchanging hyperpolarized ligand such as benzylamine can be efficiently detected using a ligand concentration that is higher than the protein concentration because of the fast relaxation rate of the bound form.^15^ Nevertheless, for the observation of ligand binding, lower ligand and protein concentrations in the approximately mM and μM range, as shown in Figure 2, are preferred. For the high-resolution experiment, these concentrations would easily be achievable with ^13^C labeling, which would increase the intensity of the signal by 100-fold. Additionally, the signal-to-noise ratio would increase 3× by using a cryoprobe, 2× by an improved filling factor of the flow cell in the detection coil, and 3× by a higher magnetic field in an 800 MHz instead of a 400 MHz NMR spectrometer.

**Figure 4:**
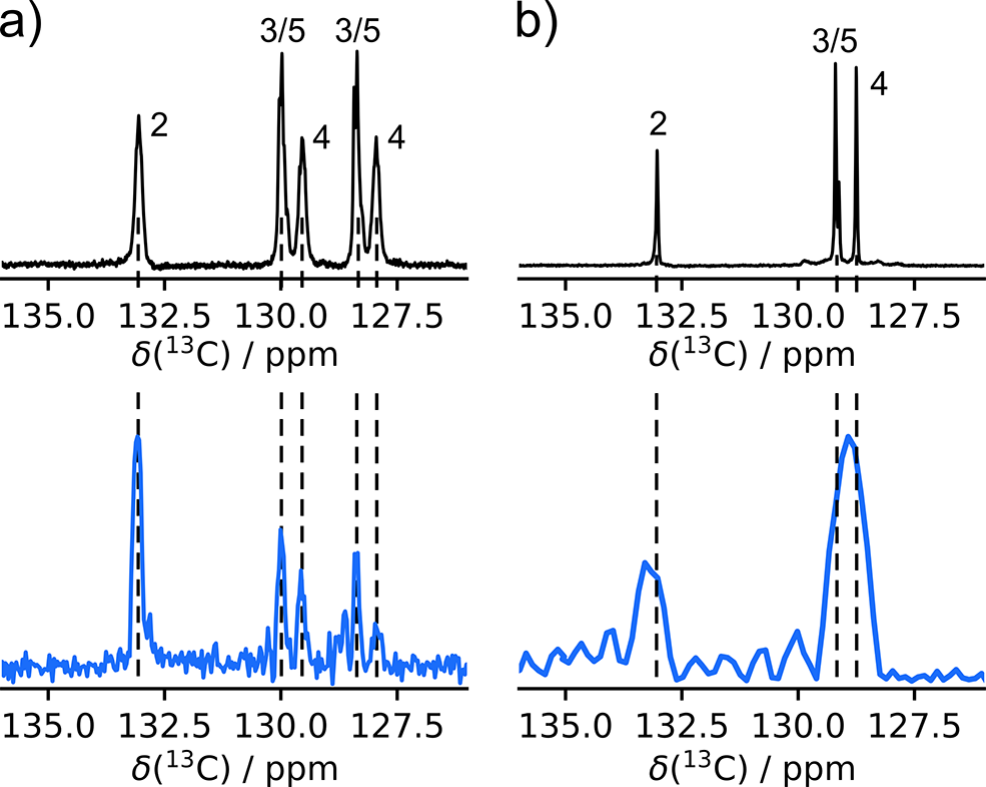
(a) Hyperpolarized ^13^C NMR spectrum of benzylamine without ^1^H decoupling (top) and sum of the magnitude of the first 16 echoes from the ultrafast chemical shift-*R*_2_ spectra with *T*_E_ = 20 ms and 40.3 mM concentration (bottom). (b) Hyperpolarized ^13^C NMR spectrum with ^1^H decoupling (top) and sum of first 16 echoes from the ultrafast spectra with *T*_E_ = 5 ms and 6.08 mM concentration (bottom).

The spectra may be further improved by ^1^H decoupling with the optional refocusing pulse in Figure 1.^27^ Here, the doublets for C3/5 and C4 collapsed, and the signal-to-noise ratio improved to 9.99 and 7.99 for the first echo without and with protein (Figure 4b). The relaxation rates remained unchanged within error limits (Figure S5 and Table S2).

The resolution of the spectra is not affected by reducing the echo times, which is required to access molecular dynamics on a millisecond time scale. The minimum echo time is predominantly governed by the maximum speed of digitization (Figure S8a).

In summary, we demonstrated the measurement of *R*_2_ relaxation of hyperpolarized ^13^C spins with ultrafast spatial encoding and a readout of chemical shifts. The ultrafast encoding beats the sampling time-imposed resolution limit of conventional NMR. The binding of a ligand, benzylamine, to the protein trypsin could be proven from a significant change in multiple observed relaxation rates. Combined with nuclear spin hyperpolarization, new relaxometry applications become possible, such as the measurement of dynamics and its role in the function of biological molecules, exchange processes in molecular interactions, and fast chemical reactions.

## Supporting Information Available

Experimental methods; DNP signal enhancement; chemical shift calibration; spectral resolution; *R*_2_ measurements; diffusion effects; effect of decoupling; selection of echo time. (PDF)
