# Supplementary material for: Measuring Protein–Ligand Binding by Hyperpolarized Ultrafast NMR
Source: J Am Chem Soc. 2024 Feb 19;146(8):5063–6. doi: 10.1021/jacs.3c14359 (PMC10910566; doi:10.1021/jacs.3c14359)
Supplement: Supplementary file 1 — ja3c14359_si_001.pdf [file ja3c14359_si_001.pdf]

Supporting Information for

Measuring Protein-Ligand Binding by Hyperpolarized Ultrafast NMR

Chang Qi,[a] Otto Mankinen,*[b] Ville-Veikko Telkki,[b] and Christian Hilty*[a]

[a] Chemistry Department, Texas A&M University, College Station, TX 77845, USA

[b] NMR Research Unit, Faculty of Science, University of Oulu, 90014 Oulu, Finland

* Corresponding authors. E-mail: C. Hilty. chilty@tamu.edu, O. Mankinen. otto.mankinen@oulu.fi

Table of Contents

Experimental Methods....2
DNP Hyperpolarization....2
Ultrafast NMR Experiment....3
DNP Signal Enhancement....4
Chemical Shift Calibration....4
Spectral Resolution....5
R_2 Measurements....6
Diffusion Effects....8
Effect of Decoupling....9
Selection of Echo Time....11
References....11

Experimental Methods

DNP Hyperpolarization

The sample for DNP polarization consisted of 8.06 M benzylamine (Sigma Aldrich, St. Louis, MO) with 15 mM OX063 (Oxford Instruments, Abingdon, UK) and 1 mM Gd-DTPA (GE Healthcare, Chicago, IL). The sample was glass-forming without the addition of other components. It was prepared by adding stock solutions for OX063 (150 mM in H_2O) and Gd-DTPA (50 mM in H_2O) to pure benzylamine. For the DNP experiment, 1.5 μL of the sample was loaded in a HyperSense polarizer (Oxford Instruments, Abingdon, UK) operating at a magnetic field of 3.35 T. The volume of the DNP sample was increased to 20 μL in the experiment with longer T_E (Figure 4a). The ^{13}C spins of the sample were polarized by irradiating 60 mW of 93.974 GHz microwaves for 3 hours at a temperature of 1.4 K. After hyperpolarization, the sample was dissolved by preheated Tris·HCl buffer, taken into a sample loop and transferred rapidly to a 3D printed flow cell of 240 μL volume (Figure S1). The flow cell was printed using a clear printer resin (Anycubic). Details of the cell will be described in a subsequent publication. The cell was placed in a 5 mm Triple Resonance Inverse (TXI) probe in a 400 MHz NMR magnet (Bruker Biospin, Billerica, MA). It was connected to a liquid injection system using poly ether ether ketone (PEEK) tubing of 0.02 inch inner diameter. A second sample loop containing 0.4 mL protein sample was connected to the flow cell. In the experiment without protein, the protein sample was replaced by Tris buffer. The injection of sample in both loops was achieved using water driven by high pressure syringe pumps.[1] The pressure and timing of the injection of the protein solution was controlled separately from the DNP sample. The injection parameters were optimized to achieve the maximum concentration of both hyperpolarized ligand and protein delivered to the flow cell. The flow rates of pressurized water that drive the DNP sample and protein were set to 100 mL/min and 180 mL/min, respectively. The corresponding injection times were 980 ms and 800 ms, with simultaneous end time. A 1.5 s delay was included after injection for stabilization of the sample.

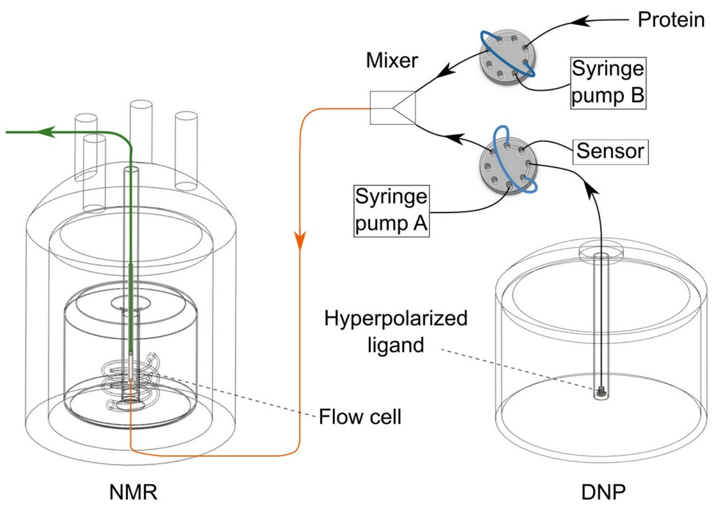

Figure S1. *Setup for the DNP experiments. Hyperpolarization occurs in the DNP polarizer (right), after which the sample is dissolved using heated solvent and automatically transferred into an injection loop. The non-hyperpolarized protein sample is pre-loaded in a second loop. Two syringe pumps push*

the samples through a mixer into a flow cell installed in the NMR spectrometer, with a final sample volume of 240 µL (left). The flow is stopped by switching off the injection valves.

The buffer contains 40 mM Tris and 10 mM $CaCl_2$. Its pH was adjusted to 7.6 before mixing, to reach final pH of 8 after dissolving the benzylamine. The same buffer with pH adjusted to 1.1 was used for the experiment with the DNP sample of larger volume. The lower pH of the buffer was to counteract the limited buffering capacity facing the mixing with pure benzylamine. The required initial pH was determined experimentally by dissolving benzylamine in buffers at an array of pH values. The buffer that resulted in a final pH of 8 was chosen for the DNP experiments. The pH resulting from the sample diluted in the dissolution system was further verified by injecting a sample into a tube instead of the flow cell, from where the pH was measured.

For the experiments with protein, a solution containing 139 µM trypsin (AMERSCO, Solon, OH) and 200 µM sodium trimethylsilylpropanesulfonate (DSS; Cambridge Isotope Laboratories, Andover, MA) in the buffer adjusted to pH 8 was preloaded in a second sample loop. After injection, the integral of the DSS methyl signal was measured and compared to that of a sample with 2 mM DSS to calculate the dilution factor for the protein sample. The pH of the Tris buffer loaded in the second sample loop instead of protein was adjusted to 8.

Ultrafast NMR Experiment

The ultrafast NMR pulse sequence to measure the R_2 relaxation rates of ^{13}C spins is shown in Figure 1 in the main text. A constant-time chemical shift encoding block started by applying a 21 µs hard $\pi/2$ pulse on the carbon channel, followed by two adiabatic Chirp pulses of 5 ms duration, 15 kHz sweep width, and field strength γB_1 = 1.55 kHz, accompanied by the bipolar encoding gradients G_S (6.8 G/cm). The selection of constant time encoding prevents an unequal signal reduction due to diffusion during the encoding time.[2] The readout block started after the dephasing gradient G_D (11.6 G/cm, 3.09 ms) to dephase the spins. The data acquisition took place with the readout gradient G_R (11.6 G/cm, 6.18 ms) applied during the CPMG loop in between the hard π pulses. The CPMG loop included n = 512 π pulses and echoes, and 512 complex data points were acquired in each echo. The echo time was 2Δ = 7 ms. The dwell time for the data acquisition was $5 \cdot 10^{-6}$ s per real or imaginary point. In the experiment shown in Figure 4a, the duration of Chirp pulses in the encoding block was extended to 20 ms to provide an increased resolution of the encoded chemical shifts. In the experiments that used decoupling, the decoupling was achieved by either applying a π pulse on the ^1H channel in the middle of the two Chirp pulses or both a π pulse during encoding and an adiabatic pulse (WURST pulse of 700 µs duration, 100 kHz sweep width, γB_1 = 10.66 kHz) during acquisition. The adiabatic pulse started simultaneously with the dephasing gradient and ended after the acquisition finished.

The NMR data was recorded using a 400 MHz NMR spectrometer (Bruker Biospin) at a temperature of 298 K. The raw data files containing 512 x 512 points were read using Python (Python Software Foundation, https://www.python.org) and converted to magnitude. Every second echo was inverted in time to correct for the chemical shift inversion because of the π inversion pulses between echoes. To compensate for the delay of the digital filter applied by the spectrometer, which introduced a nominal group delay of 68 points, several points were removed from the beginning of echo. The number of cut

off points was adjusted to 72, until the peaks from each echo aligned. The remaining number of complex points in each echo was 512-72 = 440.

For each peak to be integrated, the points in the selected region that includes the peak were summed up. The relaxation rates were obtained by fitting these integrals from each echo using the equation $S = S_0 \cdot \exp(-R_2 \cdot t) + c$.

DNP Signal Enhancement

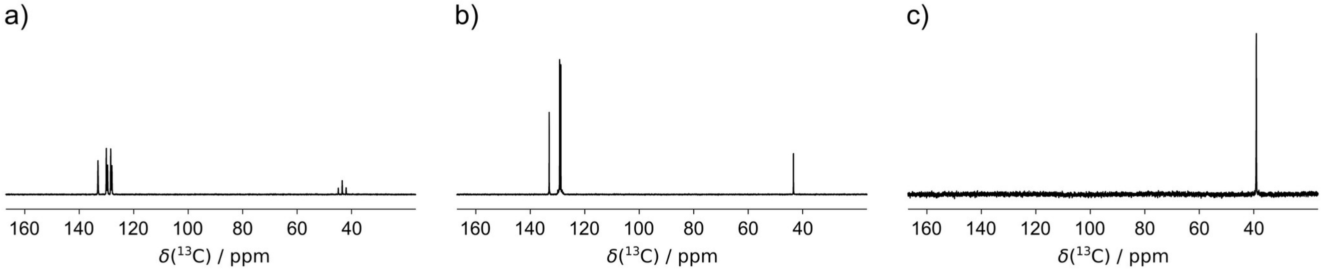

Figure S2. *a) ^{13}C NMR spectrum of 6.81 mM hyperpolarized benzylamine measured without decoupling. b) ^{13}C NMR spectrum of 6.15 mM hyperpolarized benzylamine with decoupling. c) Non-hyperpolarized ^{13}C NMR spectrum of 1M DMSO with decoupling during acquisition, measured with 128 scans. The spectra in (a) and (b) are shown with the same scale; the spectrum in (c) is scaled down by a factor of 2. All spectra were measured in the flow cell. Hyperpolarized samples were injected as described. The non-hyperpolarized sample of DMSO was loaded in a syringe and manually pushed into the flow cell.*

The ^{13}C signal enhancement in the DNP experiments was determined by comparing the integral of each peak in the spectra of hyperpolarized benzylamine (Figure S2a and b) to that of the decoupled DMSO peak in a non-hyperpolarized experiment (Figure S2c). In the spectrum without decoupling (Figure S2a), the calculated signal enhancement values for the peaks C2, as well as left and right doublet of C3/4/5 at 133.1 ppm, 129.7 ppm, and 127.9 ppm are 5800, 4700 and 4500, respectively. In the spectrum with decoupling (Figure S2b), the calculated signal enhancement values for the peaks C2 and C3/4/5 at 133.1 ppm and 128.9 ppm are 6300 and 4700, respectively.

Chemical Shift Calibration

In the ultrafast experiment, the time-axis of the acquired echoes, represented by the data point number, can be converted to a chemical shift axis. The conversion factor depends on the gradient strength and other parameters, as shown in the equation[3]

$$\Omega = \frac{G_R \cdot \Delta O \cdot dw}{2 G_S \cdot T_E} \cdot \qquad \text{(S1)}$$

Here, G_R = 0.12 Tm^{-1} and G_S = 0.068 Tm^{-1} are the readout and encoding gradient strengths, respectively, ΔO = 15 kHz is the Chrip pulse bandwidth, and dw = 5 μs/pt is the dwell time. T_E is the duration of the encoding gradient. When T_E = 5 ms, as used for most of the experiments described in the text, the conversion factor is Ω = 12.75 Hz/pt. For the data in Figure 4a in the main text, where the

T_E was increased to 20 ms, the conversion factor is 3.19 Hz/pt. If the frequencies are converted to chemical shifts, the conversion factor becomes δ = 0.1266 ppm/pt for T_E = 5 ms and δ = 0.0317 ppm/pt for T_E = 20 ms.

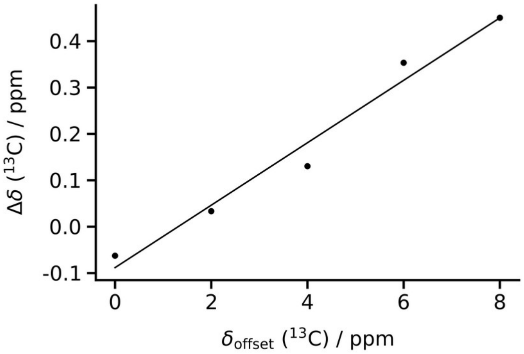

Figure S3. *The horizontal axis represents the* ^{13}*C chemical shift offset,* δ_{offset}*, between the center of the acquired data and the chemical shift observed in a 1D* ^{13}*C NMR experiment. The vertical axis represents for the* ^{13}*C chemical shift difference,* $\Delta\delta$*, between the* ^{13}*C chemical shift measured in the ultrafast experiment or in a 1D* ^{13}*C NMR experiment. In this figure, the left doublet of non-hyperpolarized* $CHCl_3$ *was measured. Linear fitting equation of the chemical shift offset is* $\Delta\delta = 0.0673(\delta_{\mathrm{offset}}) - 0.0886$, $R^2 = 0.9741$.

Experimentally, the peak positions were found to deviate somewhat from the positions expected from Equation S1. Such deviations may arise for example due to small errors in the magnitude of the applied gradients. An experimental calibration was performed to compensate for any such imperfections. The calibration was based on the echo position measured from a test sample. The same pulse sequence as in the actual DNP experiments was used in order to capture the effects of potential sources of errors such as gradient ramping times, offsets, *etc*. In the calibration measurement shown in Figure S3, a series of non-hyperpolarized experiments with different frequency offsets were performed on a $CHCl_3$ sample. For each experiment, the deviation of the peak position from the known chemical shift was recorded. The deviation was fitted to a linear equation, which served as a calibration curve. To perform the calibration for an ultrafast experiment, the chemical shift of each point was calculated as $\delta' = \delta + \Delta\delta$, where the chemical shift offset $\Delta\delta$ is from the equation fitted from Figure S3. Here, the calibration resulted in a stretching of the chemical shift axis of 0.4 ppm over a range of 8 ppm, *i.e.* by 5 %.

Spectral Resolution

In the following, the resolution in the ultrafast experiments is calculated and compared to the resolution achieved with a conventional Carr-Purcell-Meiboom-Gill (CPMG) experiment. The peak width in the ultrafast experiment depends on the duration of the encoding gradient and is notably independent of the echo time in the acquisition, as shown in Ref. 4. The full width at half height, $\Delta\nu_{1/2}$, is given by

$$\Delta\nu_{1/2} = 0.6\,\Delta\nu_{full} = \frac{0.6}{2T_E} \quad \text{(S2)}$$

Here, $\Delta\nu_{\mathrm{full}}$ is the width at the base of the sinc shaped peak (i.e. distance between the points corresponding to the first zero crossings of the sinc function), and the factor of 0.6 is the conversion to

the width at half height. In an ultrafast R_2 experiment as in Figure 2a (middle and bottom), $\Delta\nu_{1/2}$ calculated from Eq. S2 is 60 Hz. This value closely corresponds to a line width at half height of 63 Hz (or 0.63 ppm) for the C2 signal in Figure 2a (middle and bottom). If the duration of the encoding gradient is increased from 5 ms to 20 ms, the calculated $\Delta\nu_{1/2}$ reduces to 15 Hz. $\Delta\nu_{1/2}$ = 25 Hz was measured from C2 peak in Figure 4a. With a longer encoding time, the measured $\Delta\nu_{1/2}$ is higher than the calculated value.[4]

For comparison, the digital resolution of a conventional CPMG experiment, where each echo is directly Fourier transformed, would be

$$res = \frac{1}{2\Delta} \quad \text{(S3)}$$

With the same 2Δ = 7 ms echo time, the resolution calculated from Eq. S3 is 143 Hz. This resolution is a lower limit for the actual line width that may be observed in a conventional CPMG experiment. Under the assumption that the NMR signal does not decay, the line shape after Fourier transform is a sinc function, specifically of the form $\sin(2\pi af)/f$, where a is the half-echo time and f the frequency. For the echo time of 7 ms (a = 3.5 ms), the calculated full width at half height of this function is 172 Hz. As a result, despite that the ultrafast R_2 experiments were measured with the same echo time, the ultrafast experiment with 5 ms encoding gradient provides a resolution that is increased by a factor of > 2.5. The experiment with 20 ms encoding gradient shows a maximum theoretical improvement of 11-fold, of which an improvement of 7-fold was realized in the experiment.

The data in Figure 4a in the main text illustrates the increase in the resolution by increasing T_E from 5 ms to 20 ms. The peak width at half height was reduced to 25 Hz, as described in the main text, concomitant with a reduction in the signal sensitivity of the experiment due to relaxation and diffusion effects during the extended spatial encoding period. The final concentration of the benzylamine sample was increased from 6.54 mM to 40.3 mM in order to obtain a sufficient amount of signal. With the high benzylamine concentration, the pH of the dissolution buffer was adjusted to 1.1 to maintain the final pH at ~8. Under these conditions, elevated relaxation rates were observed, which were probably caused by paramagnetic ions dissolved from the DNP polarizer by the heated acid. These rates were not further analyzed. In non-hyperpolarized experiments, where T_E was set to 5 ms or 20 ms, R_2 for carbon spins from a pure benzylamine sample indeed did not change significantly.

R_2 Measurements

Each hyperpolarized ultrafast experiment without or with trypsin was repeated three times. In the presence of trypsin, an R_2 increase was observed. One complete data set at each condition is shown in Figure 3 in the main text. Figure S4 contains two additional data sets at each condition.

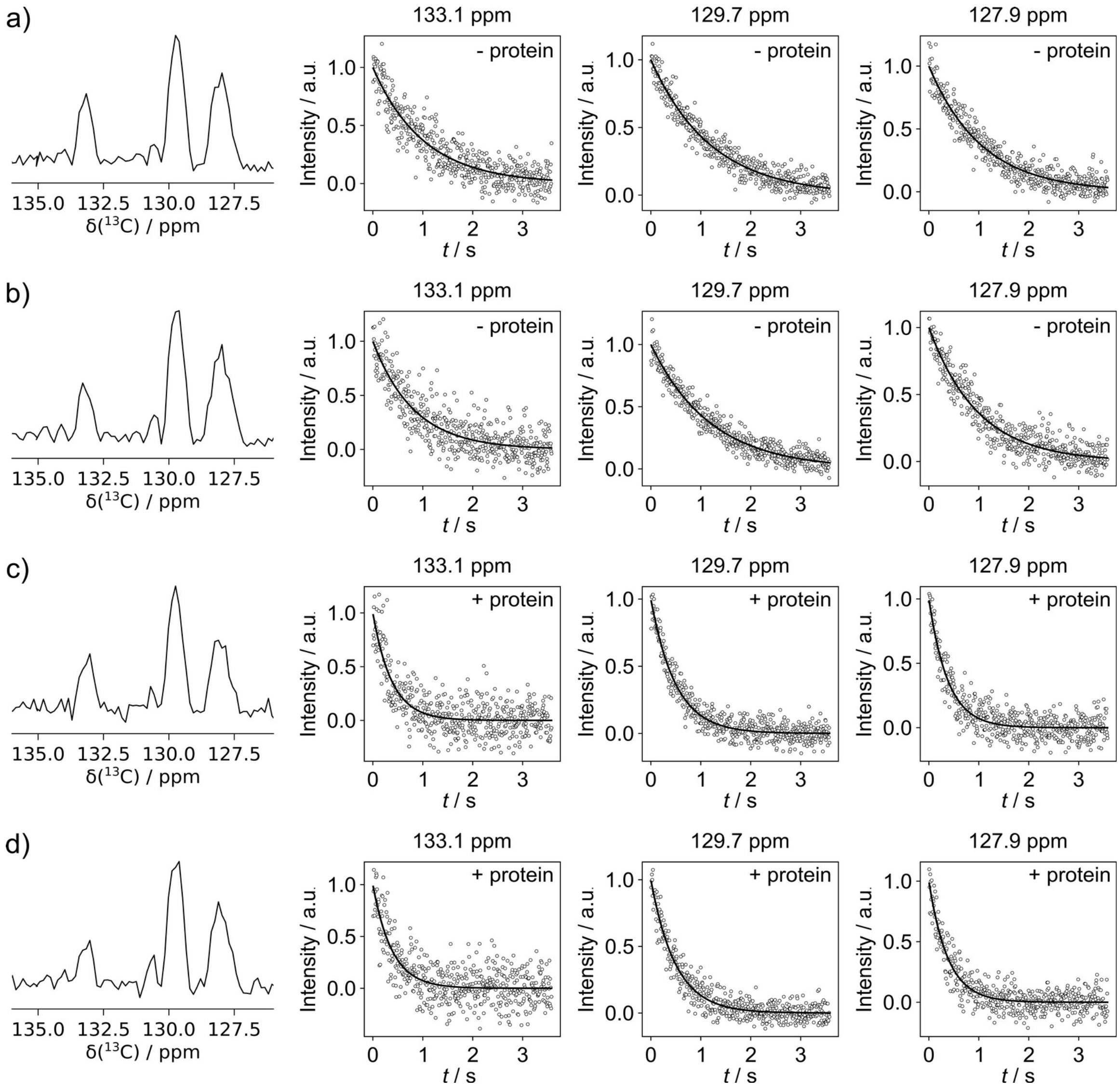

Figure S4. *The sum of the magnitude of the first 16 echoes (left) and the signal decays for carbon spins (right) obtained from the ultrafast chemical shift-R_2 spectra of benzylamine without ^{1}H decoupling in the absence (a and b) and presence (c and d) of protein. The fitted R_2 and standard deviation are summarized in Table S1.*

Table S1. *Relaxation rates for carbon peaks at 133.1 ppm, 129.7 ppm, and 127.9 ppm of hyperpolarized benzylamine in the absence and presence of trypsin. The data sets are graphed in the figures indicated in the first column. Standard errors from R_2 fitting are indicated.*

No.	[Benzylamine]$_0$ / mM	[Trypsin]$_0$ / µM	R_2 for peak at 133.1 ppm	R_2 for peak at 129.7 ppm	R_2 for peak at 127.9 ppm
Figure S4a	5.80	—	0.98 ± 0.06	0.82 ± 0.03	0.94 ± 0.04
Figure S4b	5.82	—	1.22 ± 0.08	0.84 ± 0.03	1.02 ± 0.04

Figure 2a middle	6.54	—	0.83 ± 0.05	0.86 ± 0.03	0.98 ± 0.03
Figure S4c	6.19	26.2	2.65 ± 0.18	2.01 ± 0.06	2.63 ± 0.11
Figure S4d	6.15	28.9	2.61 ± 0.19	2.02 ± 0.06	2.64 ± 0.11
Figure 2a bottom	6.69	32.3	1.90 ± 0.07	1.88 ± 0.05	2.73 ± 0.11

Diffusion Effects

This section evaluates the signal changes in the CPMG loop due to the diffusion effects caused by the readout gradient. The diffusion effect on R_2 decay, R_{diff}, is estimated from the Stejskal–Tanner equation.[5]

The signal decay in the CPMG loop due to T_2 relaxation and diffusion effects is[5,6]

$$S = S_0 \exp(-R_2 t)\exp(-b_{uf} D \cdot n) . \quad \text{(S4)}$$

Here, R_2 is the transverse relaxation rate, D is the self-diffusion coefficient of the DNP polarized analyte, n is the number of echoes, and b_{uf} refers to the b-value resulting from the readout gradient in every echo calculated by

$$b_{uf} = \gamma^2 G_R^2 \delta^2 \left(\Delta - \frac{\delta}{3} \right), \quad \text{(S5)}$$

where $\gamma = 67.3 \cdot 10^6\ \mathrm{s^{-1}T^{-1}}$ is the ^{13}C gyromagnetic ratio, $G_R = 0.12\ \mathrm{Tm^{-1}}$ is the readout gradient strength, $\delta = 3.09$ ms is half of the duration of readout gradient G_R, and $\Delta = 3.5$ ms is half of the echo time.

The observed R_2 relaxation rate is derived from Eqs. S4 ans S5 by substituting the number of echoes, $n = t/(2\Delta)$, where t is the total acquisition time:

$$R_{2,obs} = R_2 + b_{uf} D / (2\Delta) \cdot \quad \text{(S6)}$$

R_2 is the intrinsic relaxation rate in the absence of gradients from Eq. S4. The relaxation contribution due to the diffusion effect is

$$R_{diff} = b_{uf} D / (2\Delta) \quad \text{(S7)}$$

With the experimental parameters, $R_{diff} = 2.2 \cdot 10^8\ \mathrm{m^{-2}} \cdot D$. To determine the self-diffusion coefficient for benzylamine, the D for water sample was measured and compared to the literature value for the calibration of gradient strength. The D for benzylamine was measured as $0.82 \cdot 10^{-9}\ \mathrm{m^2/s}$, resulting in a diffusion contribution R_{diff} on the order of 0.18 $\mathrm{s^{-1}}$.

Effect of Decoupling

In addition to the undecoupled spectra, relaxation rates were measured with the application of two decoupling methods. These include a hard π pulse applied during the encoding sequence (Figures 4b and S5 and Table S2), or the hard π pulse applied during the encoding sequence in combination with an adiabatic decoupling sequence during the signal acquisition period (Figures S6 and S7 and Table S3). The adiabatic decoupling sequence did not improve the signal-to-noise ratio, however, induced faster relaxation.

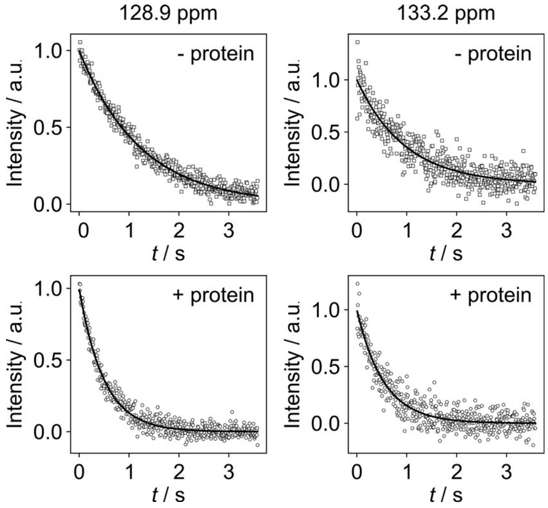

Figure S5. *Signal decays obtained by the ultrafast sequence with decoupling by applying a hard π pulse on the* ^{1}H *channel during encoding only without (top) and with (bottom) protein. Corresponding spectra are shown in Figure 4b in the main text.*

Table S2. *Relaxation rates for carbon peaks at 128.9 ppm and 133.2 ppm of hyperpolarized benzylamine in the absence and presence of trypsin.* R_2 *was measured using the decoupled sequence with a π pulse on the* ^{1}H *channel during encoding. Standard errors from* R_2 *fitting are indicated.*

$[\text{Benzylamine}]_0$ / mM	$[\text{Trypsin}]_0$ / µM	R_2 for peak at 128.9 ppm	R_2 for peak at 133.2 ppm
6.08	—	0.81 ± 0.02	1.04 ± 0.05
6.29	38.4	2.03 ± 0.04	1.86 ± 0.07

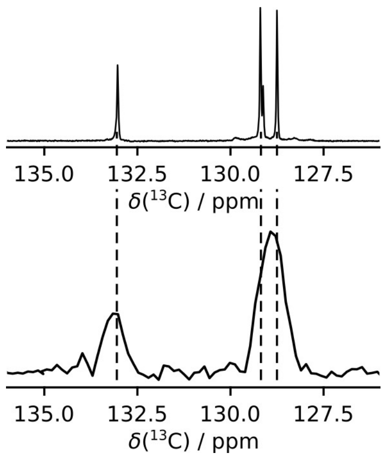

Figure S6. *Hyperpolarized* [13]*C NMR spectrum of benzylamine with decoupling (top) and the sum of the magnitude of the first 16 echoes from the ultrafast chemical shift-*R_2 *spectra of benzylamine in the absence of protein (bottom). The ultrafast signal was measured with a π pulse on the* [1]*H channel during encoding and an adiabatic decoupling pulse during acquisition. Dashed lines indicate the chemical shift alignment.*

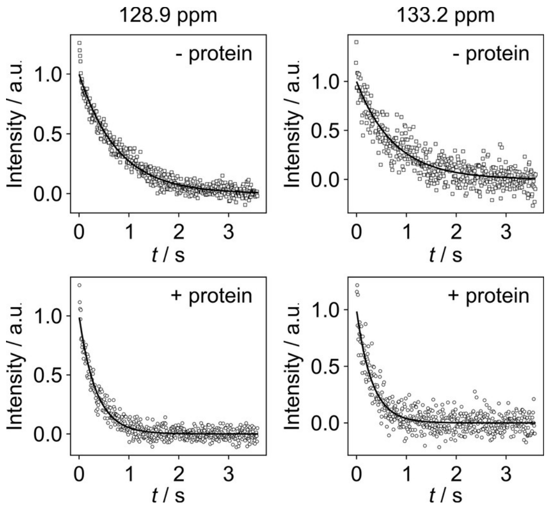

Figure S7. *Signal decays obtained by the ultrafast sequence with a π pulse on the* [1]*H channel during encoding and an adiabatic decoupling pulse during acquisition without (top) and with (bottom) protein.*

Table S3. *Relaxation rates for carbon peaks at 128.9 ppm and 133.2 ppm of hyperpolarized benzylamine in the absence and presence of trypsin.* R_2 *was measured using the decoupled sequence with a π pulse on the* [1]*H channel during encoding and an adiabatic decoupling pulse during acquisition. Standard errors from* R_2 *fitting are indicated.*

$[\text{Benzylamine}]_0$ / mM	$[\text{Trypsin}]_0$ / µM	R_2 for peak at 128.9 ppm	R_2 for peak at 133.2 ppm
6.15	—	1.30 ± 0.03	1.33 ± 0.06
6.37	34.5	2.93 ± 0.07	3.25 ± 0.14

Selection of Echo Time

A suitable echo time can be determined before the measurement to cover the chemical shift range that includes all peaks. Figure S8 illustrates two different spectral widths obtained with two echo times. The figures show all data points measured with an echo time of 2.7 ms and 7 ms. According to Eq. S1, the frequency range of the observation window is (n-72)*0.1266 ppm/pt, where n is the number of points obtained in each echo, and 72 points were removed in processing. The calculated chemical shift range is 7.1 ppm for Figure S8a and 55.7 ppm for Figure S8b. Therefore, to observe the peaks of this sample within 6 ppm, the lower limit of the useful echo time is determined to be about 2.7 ms.

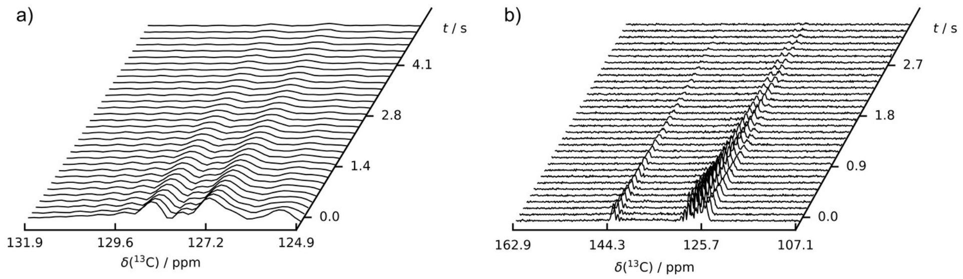

Figure S8. *Ultrafast R_2 measurement of a pure benzylamine sample in non-hyperpolarized experiments. ^{13}C chemical shifts are different for pure benzylamine and the DNP sample. a) 128 complex points were acquired in each echo with an echo time of 2.7 ms. The sum of the magnitude of every 64 echos is plotted against time. 56 data points (128-72=56) are shown in this figure. b) 512 complex points were acquired in each echo with an echo time of 7 ms. The sum of the magnitude of every 16 echos is plotted against time. 440 data points (512-72=440) are shown in this figure.*

References

(1) Chen, H.-Y.; Hilty, C. Implementation and Characterization of Flow Injection in Dissolution Dynamic Nuclear Polarization NMR Spectroscopy. *ChemPhysChem* **2015**, *16* (12), 2646–2652.
(2) Giraudeau, P.; Akoka, S. Sources of Sensitivity Losses in Ultrafast 2D NMR. *Journal of Magnetic Resonance* **2008**, *192* (1), 151–158.
(3) Tal, A.; Frydman, L. Single-Scan Multidimensional Magnetic Resonance. *Progress in Nuclear Magnetic Resonance Spectroscopy* **2010**, *57* (3), 241–292.
(4) Giraudeau, P.; Akoka, S. Resolution and Sensitivity Aspects of Ultrafast J-Resolved 2D NMR Spectra. *Journal of Magnetic Resonance* **2008**, *190* (2), 339–345.
(5) Stejskal, E. O.; Tanner, J. E. Spin Diffusion Measurements: Spin Echoes in the Presence of a Time-Dependent Field Gradient. *The Journal of Chemical Physics* **1965**, *42* (1), 288–292.
(6) Shrot, Y.; Frydman, L. The Effects of Molecular Diffusion in Ultrafast Two-Dimensional Nuclear Magnetic Resonance. *J Chem Phys* **2008**, *128* (16), 164513.
